# Effectiveness of bladder filling control during online MR-guided adaptive radiotherapy for rectal cancer

**DOI:** 10.1186/s13014-023-02315-3

**Published:** 2023-08-17

**Authors:** Xi Feng, Bin Tang, Xinghong Yao, Min Liu, Xiongfei Liao, Ke Yuan, Qian Peng, Lucia Clara Orlandini

**Affiliations:** 1 Department of Radiation Oncology, Sichuan Cancer Hospital & Institute, Affiliated Cancer Hospital of University of Electronic Science and Technology of China, Chengdu, China; 2 Radiation Oncology Key Laboratory of Sichuan Province, Sichuan Clinical Research Center for Cancer, Sichuan Cancer Center, Chengdu, China; 3 Institute of Nuclear Technology and Automation Engineering, Chengdu University of Technology, Chengdu, China

**Keywords:** Radiotherapy, MR-Linac, Rectum, Adaptive radiotherapy, Magnetic resonance imaging, Bladder control

## Abstract

**Background:**

Magnetic resonance-guided adaptive radiotherapy (MRgART) treatment sessions at MR-Linac are time-consuming and changes in organs at risk volumes can impact the treatment dosimetry. This study aims to evaluate the feasibility to control bladder filling during the rectum MRgART online session and its effectiveness on plan dosimetry.

**Methods:**

A total of 109 online adaptive sessions of 24 rectum cancer patients treated at Unity 1.5 T MR-Linac with a short course radiotherapy (25 Gy, 5 Gy × 5) for whom the adaptive plan was optimized and recalculated online based on the daily magnetic resonance imaging (MRI) were analysed. Patients were fitted with a bladder catheter to control bladder filling; the bladder is emptied and then partially filled with a known amount of saline at the beginning and end of the online session. A first MRI (${\mathrm{MRI}}_{\mathrm{i}}$) acquired at the beginning of the session was used for plan adaptation and the second (${\mathrm{MRI}}_{\mathrm{f}}$) was acquired while approving the adapted plan and rigidly registered with the first to ensure the appropriateness of the isodoses on the ongoing delivery treatment. For each fraction, the time interval between the two MRIs and potential bladder changes were assessed with independent metrics, and the impact on the plan dosimetry was evaluated by comparing target and organs at risk dose volume histogram cut-off points of the plan adapted on ${\mathrm{MRI}}_{\mathrm{i}}$ and recalculated on ${\mathrm{MRI}}_{\mathrm{f}}$.

**Results:**

Median bladder volume variations, DSC, and HD of 8.17%, 0.922, and 2.92 mm were registered within a median time of 38 min between ${\mathrm{MRI}}_{\mathrm{i}}$ and ${\mathrm{MRI}}_{\mathrm{f}}$; dosimetric differences < 0.65% were registered for target coverage, and < 0.5% for bladder, small bowel and femoral heads constraints, with a *p* value > 0.05.

**Conclusion:**

The use of a bladder filling control procedure can help ensure the dosimetric accuracy of the online adapted treatment delivered.

## Background

Neoadjuvant chemo-radiotherapy (CRT) plays an important role in the multidisciplinary treatment of rectal cancer [1], mainly aiming to reduce local recurrence rates and reduce the stage of the tumour before surgery. The pathological complete response (pCR) rate after CRT for locally advanced rectal cancer (LARC) set around 15%, as reported in several trials [1], is one of the reasons why attempts are being made to find strategies to increase patients achieving a clinical complete response. Between these strategies, a safe dose escalation [2, 3] by conforming the dose as close as possible to the target, reducing planning target volume (PTV) margins. This objective is somewhat hampered by geometric uncertainties not only in delineation but also due to inter- and intra-fraction anatomical variations. To account for these uncertainties in clinical practice, the clinical target volume (CTV) is expanded to PTV, and in this expansion, some parts of the organs at risk (OAR), such as the bladder and small intestines may be encompassed leading to high doses to the OARs and consequent toxicity [3–5].

Recently, adaptive radiotherapy can benefit from magnetic resonance linear accelerators (MR-Linacs), i.e. linear accelerator that integrate a magnetic resonance imaging (MRI) scanner [6]. Adaptive radiotherapy guided by MRI images becomes therefore more effective in all those treatments where soft tissue visualisation is crucial and also offers the possibility of online adaptation of the dose distribution according to the daily anatomy of the patient [7]. This dual advantage, i.e. the possibility of having an MR imaging more suitable for visualising soft tissue than the cone beam computed tomography in use on all conventional modern accelerators, and of being able to use it to adapt the patient's anatomical contours in the same treatment session by recalculating the plan, makes this technology unique and explains the great interest that has arisen and the ongoing researches. As a result of the daily online adaptation in magnetic resonance-guided adaptive radiotherapy (MRgART), it is possible to handle intrafraction anatomical variations which has been so far considered the main remaining approximations [8].

In the MRgART workflow the treatment plan is optimised and recalculated following the daily MRI; because of the lack of tissue density information needed for dose calculation, an electronic density (ED) map should be generated; this is possible by the assignment of computed tomography (CT)-based ED values to the MR image data set. To address this issue, several approaches have been developed to generate ED maps also called synthetic computed tomography (sCT) from MR imaging (MRI-based sCT), mainly the bulk density assignment, atlas-based method, the voxel-by-voxel conversion, and deep learning [9–14]. The strategy of sCT applied in Monaco (Elekta AB, Stockholm, Sweden) treatment planning system (TPS) for 1.5 T MRgRT is bulk density assignment based on the contours drawn on patient simulation CT. More specifically, during the online adapt to shape (ATS) procedure, the daily acquired MRI will be deformably registered to simulation CT, then all contours information including average ED and the priority of density assignment on CT are propagated to MRI, then sCT is generated by bulk density assignment on MRI. It is well known, that contouring is a critical step in the radiotherapy process [15].

Variability in the shape and volume of OARs with different fill states can lead to large changes in ED maps resulting in inaccurate dosimetry of the plan. During the online adaptive radiotherapy session, the targets and volumes are re-contextualised according to the daily anatomy, thus circumventing the inter-fractional variability. Nevertheless, it remains irrefutable that the treatment workflow time is very long and may affect the accuracy of the plan [7, 16]; in fact, the time of the session waiting for the adaptive plan may allow the bladder to fill and thus generate a dose inconsistency with the newly developed plan.

No specific studies on the potential dosimetric impact of bladder volume changes for rectal treatment have been found, but indirectly, the effect on target margin [8, 17, 18], on the small bowel toxicity [19]; it seems difficult to disengage the need of target margin revision from anatomical variations of the adjacent OARs including those of the bladder. The impact of bladder changes in the dosimetry of the plan has been investigated for other pelvic treatments, with tumor sites located close to the bladder; Dutta et al. [20] found that bladder filling variation in the treatment of cervix carcinoma impacts the dosimetry of the target and OARs; for the prostate treatment Roeske et al. [21] and Huang et al. [22] observed that despite strict bladder protocol, the bladder tends to change shape and position and volume and noted higher doses being delivered than initially calculated, while Smith et al. [23] found that despite varying bladder volumes, all bladder and bowel constraints were achieved. It is well known that bladder filling is the most important contributing factor to small bowel irradiation, leading to the recommendation to treat with a full bladder [24]; nevertheless, studies demonstrate that instructing patients to have a full bladder for pelvic radiotherapy results in highly variable bladder volumes at daily treatment [25].

This research aims to evaluate the effectiveness of using a bladder-filling control (BfC) procedure during MRgART sessions for rectal cancer treatments to ensure the accuracy of the dosimetry of the adapted delivered plans.

## Methods

### Patients

Twenty-four patients with intermediate or locally advanced rectal carcinoma consecutively treated with a 1.5 T MR-Linac (Unity, Elekta AB, Stockholm Sweden) from October 2021 to August 2022 were included in this retrospective study. Patients received short-course radiotherapy (SCRT; 25 Gy, 5 Gy × 5) and followed the bladder control procedure as outlined in our internal clinical guidelines. An additional cohort of 14 patients treated at Unity for cervix (6 patients) or prostate (8) who did not follow the bladder control procedure and with the bladder imaged twice during the online adaptive sessions, were included in the research with the only purpose to study the bladder filling with time. Written informed consent was obtained from all the patients, and the Institutional Ethics Committee approved this retrospective study.

### Treatment plan preparation

All patients were set up in the supine position using indexed positioning aids and KneeSTEP and FeetSTEP supports (IT-V, Innsbruck, Austria). A few days before the start of the treatment, patients underwent CT and MRI simulation scans; 1.25 slice thickness CT scan with a big bore scanner (Philips, Eindhoven, The Netherlands) and a T2-weighted simulation scan at 1.5 Tesla scanner at MR-Linac (Elekta Unity, Elekta AB, Stockholm, Sweden) were acquired consecutively the same day. CT and MR imaging were exported into a segmentation commercial software (MIM Software Inc, Cleveland Ohio, USA) and after rigid registration, experienced radiation oncologists delineated the target and OARs. The gross target volume (GTV), mesorectal CTV, CTV of the elective lymph nodes regions, and the OARs were delineated following international guidelines [26]. PTV margins were created for the mesorectum with 5 mm in all directions. The treatment plans were performed in Monaco V-5.4 TPS, using ten to twelve individual beam angles and a 3 mm dose grid for the calculation; plans were optimised to achieve the clinical goals, particularly to encompass at least 95% the PTV by the prescription dose of 25 Gy limiting as much as possible doses to OARs following international guidelines and consensus [7, 27–30]. The details of the target dosimetric criteria and OARs constraints used for plan optimisation are reported in Table 1. The reference CT plan contains all the density bulk assignment information i.e., the contours, their corresponding average ED and the priority of each contour concerning density assignment in case of contour overlaps to use on the online adaptive step where for the adaptive plan calculation the use of the MRI-based sCT will be necessary [31].

**Table 1:** Target dosimetric criteria and OARs constraints used for the plan optimization

Region of interest	Criteria
PTV	
${\text{V}}_{{95{\text{\% }}}} \left( {23.75{\text{Gy}}} \right)$	> 99%
${\text{V}}_{{107{\text{\% }}}}$ (26.75 Gy)	< 5 cc
${\text{V}}_{{110{\text{\% }}}}$ (27.50 Gy)	< 0.5 cc
Bladder	
${\text{D}}_{{15{\text{cc}}}}$	< 18.3 Gy
${\text{D}}_{{0.5{\text{cc}}}}$	< 38 Gy
Small bowel	
${\text{D}}_{{0.5{\text{cc}}}}$	< 30 Gy; < 35 Gy^a^
${\text{D}}_{{5{\text{cc}}}}$	< 25 Gy^a^
${\text{D}}_{{10{\text{cc}}}}$	< 25 Gy
Femoral heads	
${\text{D}}_{{10{\text{cc}}}}$	< 30 Gy

*PTV* planning target volume, *V*_*x*%_ volume covered by x% of the prescription dose, *D*_*ycc*_ dose received y cube centimetres

^a^The optimal value

### MR-based adaptive workflow

Daily online adaptive radiotherapy was delivered following the ATS workflow [32] consisting of a plan adaptation based on the MRI acquired in each session and optimised on the corresponding MR-based sCT. At each adaptive session, once the patient is positioned on the couch a first MRI is acquired (${\mathrm{MRI}}_{\mathrm{i}}$) and rigidly registered to the CT images of the reference plan; patient contours were adapted to the daily anatomy first using an automated deformable registration software and then manually by the radiation oncologist encharged of the patient. Once the contours perfectly fit the patient’s anatomy, an adapted plan was reoptimised following the same criteria of the reference plan; these two steps involving a recontouring and plan reoptimisation may be time-consuming; therefore, during the last steps of the plan optimization a second daily MRI (${\mathrm{MRI}}_{\mathrm{f}}$) is acquired, rigidly registered with ${\mathrm{MRI}}_{\mathrm{i}}$ on which the plan adaptation is ongoing to be sure that in this time interval, the treatment that will be delivered will be appropriate.

### Bladder filling control procedure

To stabilize the bladder filling during the MRgART sessions, the patient is fitted with a bladder catheter; at the beginning of the session and before beam delivery (before MRIi and MRIf, respectively) the urine present in the bladder is drained and the same amount of saline is instilled. The indicative amount of saline to be instilled and tolerated by the patient is determined at the simulation CT scan, considering that the patient should not feel discomfort in the bladder or have the sensation of having it too full; this amount is then validated by the radiation oncologist also on the basis of the CT images, varies between 120 and 200 mL and is used as a reference during the adaptive sessions.

### Data analysis

For each adapted session the interval time between ${\mathrm{MRI}}_{\mathrm{i}}$ and ${\mathrm{MRI}}_{\mathrm{f}}$ was registered. The volumes of the bladder delineated on ${\mathrm{MRI}}_{\mathrm{i}}$ and ${\mathrm{MRI}}_{\mathrm{f}}$ were analysed. The bladder contour on ${\mathrm{MRI}}_{\mathrm{i}}$ was delineated during the online session because part of the contour dataset used for the plan adaptation, while the bladder volume on ${\mathrm{MRI}}_{\mathrm{f}}$ was delineated retrospectively by the same radiation oncologist and for this study.

A comparison of the bladder changes in volume and shape along each adaptive session was performed. Quantitative metrics [33] were used for each patient to compare the bladder changes for each delivered fraction. We reported the Dice similarity coefficient (DSC) and the Hausdorff distance (HD). The DSC provides a measure of overlap between the bladder volumes at the beginning and end of the session, with 0 indicating no overlap and 1 indicating perfect overlap. The HD is indicative of deviations between the delineation on the surface and is particularly sensitive to local surface deviations. To evaluate the impact of the BfC procedure on the dosimetry of the delivered plan for the rectum cancer treatments, for each session, the online adapted plan optimised on ${\mathrm{MRI}}_{\mathrm{i}}$ was recalculated on ${\mathrm{MRI}}_{\mathrm{f}}$, and the target and organs at risk dose volume histogram (DVH) dosimetric parameters were compared.

## Results

A total of 109 online rectal cancer MRgART sessions (consisting of 218 MRIs) of 24 patients, namely 15 males (62.5%) and 9 females (37.5%), who underwent the BfC procedure, and a further 60 online adaptive sessions for a total of 120 MRIs of a further 14 patients treated at other tumor sites and for whom the BfC procedure was not followed, were analysed.

**Fig. 1:**
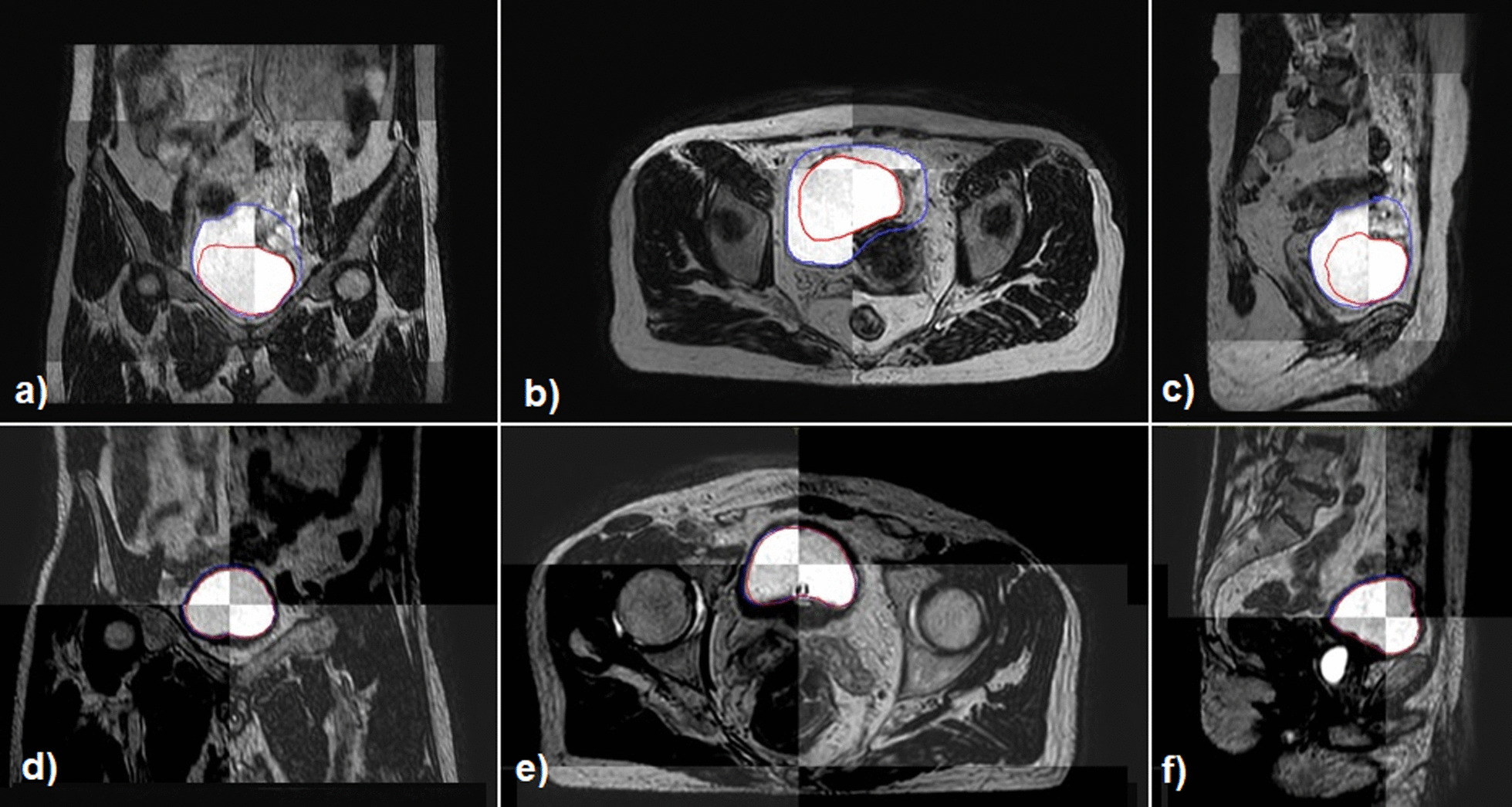
Bladders contours on MRI scans acquired at the beginning and the end of a 1.5 T MR-Linac online adaptive session and rigidly registered. Coronal, transversal and sagittal MRI planes for a representative patient not undergoing (**a**–**c**) and undergoing (**d**–**f**) the bladder filling control procedure. In red and blue the bladder contoured on the MRI acquired at the beginning and end of the session

**Fig. 2:**
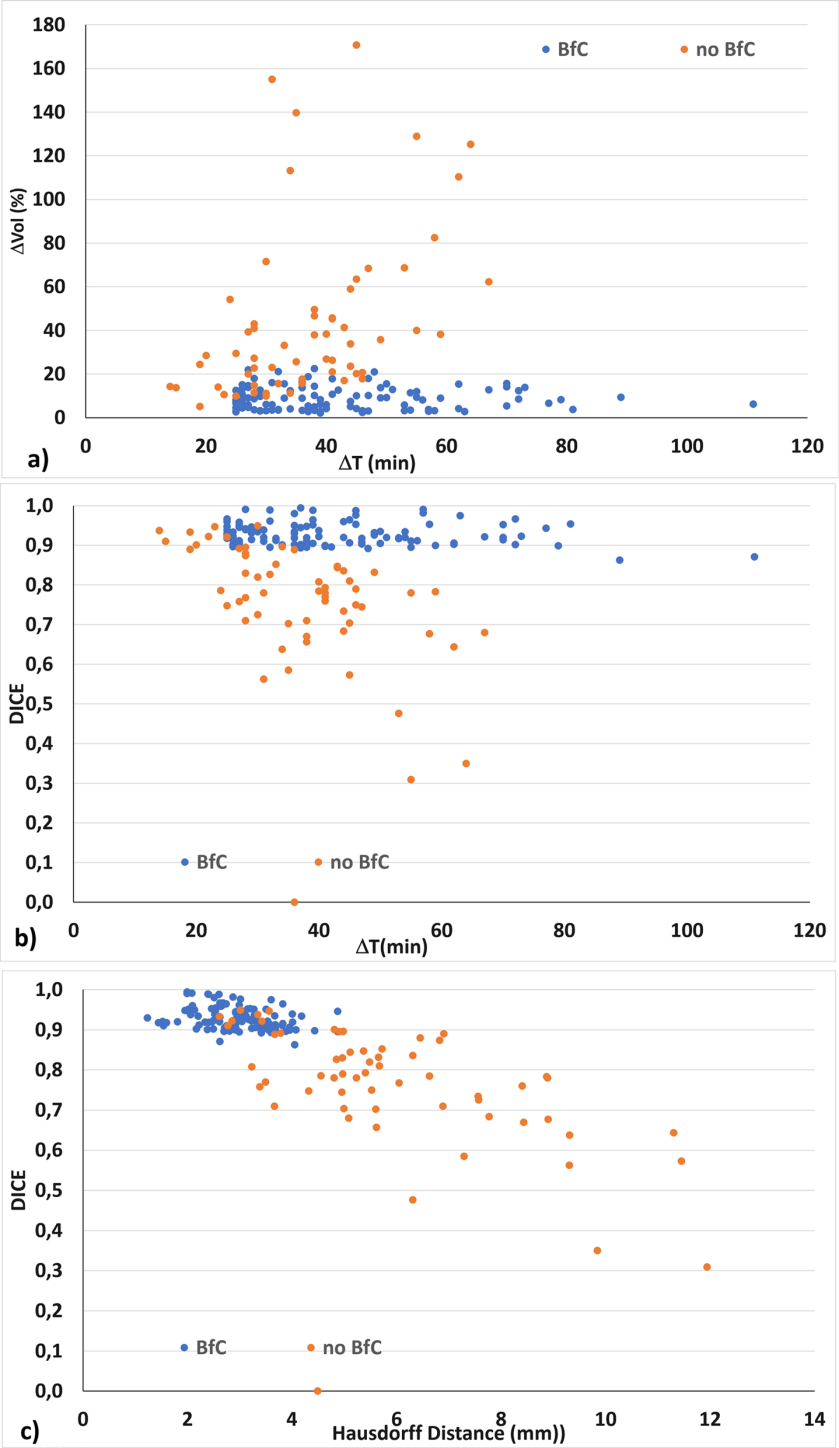
Bladder volume changes during the online adaptive session for patients following and not following the bladder filling control procedure. Bladder volume variations (**A**), and **B** Dice similarity coefficient (DSC) vs the duration of the adaptive session, and **C** metrics representative points, DSC and Hausdorff distance for each session

The variation in bladder volume with time and the results obtained for the independent metrics for patients who did and did not undergo the BfC procedure are summarised in Table 2. Figure 1 shows representative scans in the three projections from a 1.5 T MR-Linac acquired at the beginning and end of the adaptive session and rigidly registered, for a patient undergoing and not undergoing the BfC procedure. Figure 2 highlights the different behaviour of volume changes and DSC and HD metrics for sessions performed with and without BfC; bladder volume changes remain below a maximum value of 22.51% regardless of the duration of the adaptive session for BfC sessions. Similarly, Fig. 2b shows how the DSC remains close to the value of 0.90 with minimum values of 0.863 and 0.871 related to the session of 89 and 111 min, respectively; whereas, for sessions without BfC, the value depends strongly on the elapsed time. Figure 2c shows how the (HD; DSC) points remain confined in the upper left quadrant, indicating that almost all sessions with BfC have values DSC > 0.86 and HD < 4.90 while sessions without BfC are spread in all the quadrants.

**Table 2:** Bladder changes and metrics for adaptive sessions performed with and without bladder filling control procedure

	Bladder filling control	No bladder filling control
Adaptive sessions, MRI		
Number, number	109, 218	60, 120
$\Delta$Time [${\text{MRI}}_{i}$ − ${\text{MRI}}_{f}$] (min)		
Median	38	36
Range	25–111	14–67
DSC		
Median	0.922	0.783
Range	0.863–0.994	0.309–0.949
Hausdorff distance (mm)		
Median	2.92	5.44
Range	1.23–4.87	2.60–11.94
Bladder $\Delta$volume (%)		
Median	8.17	33.46
Range	2.15–22.51	5.16–170.7

*MRI* magnetic resonance imaging, *MRI*_*i*_/*MRI*_*f*_ magnetic resonance imaging at the start/end of the adaptive session, *DSC* dice similarity coefficient

For what is concerning the dosimetry of the MRgART rectum treatments the results of the adaptive session show that the target coverage and OARs constraints respect the clinical indications and are aligned with international standards [7]; moreover, the effectiveness of the BfC procedure along each session, show that the adaptive plan optimised on MRIi and recalculated on MRIf, present no statistical difference. In Table 3 are listed the results obtained.

**Table 3:** Impact of the bladder filling control on plan dosimetry of rectum cancer adaptive treatments

	Adapted plans optimized on ${\text{MRI}}_{{\text{i}}}$	Adapted plan recalculated on ${\text{MRI}}_{{\text{f}}}$	*t* student
	Reference value Median (range)	% difference	*p*
Adapted plans			
Number	109		
PTV			
Volume (cc)	648.5 (419.4–1016.3)		
$V_{{{\text{Dpre}}}}$ (%)	95.76 (95.02–96.80)	< 0.65	> 0.05
${\text{D}}_{{0.5{\text{cc}}}}$ (Gy)	26.54 (26.23–27.90)	< 0.50	> 0.05
Bladder			
Volume (cc)	154.8 (110.4–238.6)		
${\text{D}}_{{0.5{\text{cc}}}}$ (Gy)	24.54 (17.61–26.60)	< 0.50	> 0.05
${\text{D}}_{{15{\text{cc}}}}$ (Gy)	13.96 (7.41–21.02)	< 0.15	> 0.05
Small bowel			
${\text{D}}_{{0.5{\text{cc}}}}$ (Gy)	23.72 (17.32–27.55)	< 0.45	> 0.05
${\text{D}}_{{5{\text{cc}}}}$ (Gy)	19.32 (18.55–21.99)	< 0.44	> 0.05
${\text{D}}_{{10{\text{cc}}}}$ (Gy)	17.31 (15.10–18.95)	< 0.33	> 0.05
Femoral heads			
${\text{D}}_{{10{\text{cc}}}} \left( {{\text{Gy}}} \right)$	11.56 (10.51–14.61)	< 0.10	> 0.05

*PTV* planning target volume, *V*_*Dpre*_ volume in % covered by the prescription dose, *D*_*ycc*_ received y cube centimetres,* MRI*_*f*_/* MRI*_*f*_ magnetic resonance imaging at the start/end of the adaptive session

## Discussion

In the current clinical radiotherapy practice of pelvic cancers, the bladder volume and its dose constraints are used to limit radiation exposure and subsequent toxicity. Given the focus of this study on establishing a system to guarantee the invariance of the bladder volume and consequently of the target, we validated our framework to avoid changes in the shape and volume of the bladder that may contribute to an inconsistency of the target dosimetry.

In this work, we analysed the efficacy of using a procedure to maintain stable bladder filling during the MR-Linac online ATS session. The procedure in use consists for each adaptive session of emptying and refilling the bladder with the same amount of physiological solution at the beginning and in the final step of the treatment approval before the treatment delivery. In our clinical workflow, two MRIs are performed at each session: the first one is used for the adaptive plan and a second one is acquired at the last step of the treatment approval, rigidly registered with the first one to evaluate the appropriateness of the isodoses on the last acquired anatomical image.

We analysed 109 adaptive sessions, and the time interval between the first and the second MRI ranged between 25 and 111 min; in this time interval, the median changes in bladder volume changes are of 8.17%, 0.922 for DSC, and 2.92 mm for the HD. For each session, the online adapted treatment plans retrospectively recalculated on the second MRI, showed an invariability of the dosimetry of the plan with percentage differences of the target and OARs DVH dosimetric parameters less than 0.65%; results also confirmed by the t-student analysis with a *p* value > 0.05 showing no statistical differences.

At the same time, to have a comparison group for bladder behaviour during MR-Linac sessions, 60 adaptive sessions performed for other treatments, in which the bladder volume was visible, were processed. Although the time interval between the two MRIs was smaller (range 14–67 min) compared to the BfC group, the bladder changes are higher with a median value of the volume variation of 33.46%, a median value of DSC of 0.783 and HD 5.44 mm. The results obtained indicate the effectiveness of the BfC procedure in keeping unvaried bladder changes during the session.

The comparison group was introduced for the sole purpose of monitoring changes in bladder volume, position and shape over time when no BfC procedure is in place. The changes obtained were not then used to see their impact on the dosimetry of the plan as they are treatments with targets other than the rectum, and therefore beyond the scope of this work. However, the bladder changes documented for the two different patient groups were assessed using the same imaging modalities, the same MR-linac and immobilization setup, and are representative of the clinical routine. Despite the potential heterogenity of the bladder filling between patients, the different bladder behaviour obtained in the two groups reinforces the effectiveness of the BfC procedure.

Considering that the dosimetry of the target and surrounding organs at risk are influenced by each other's location and proximity, it remains of paramount importance to keep the anatomical configuration of the adaptive plans and delivery inviolate. It is worth noting that in the control group when the interval time between the two MRIs remains less than about 20 min many sessions were found to have DSC around 0.90 and HD around 3.5 mm, indicative of bladder invariability at this time point. For this purpose, the possibility to develop an artificial intelligence-based system able to ensure the automatic and reliable full delineation of the therapy volumes even if has reached remarkable results [34, 35], is not available in the MR-Linac clinical workflow. The AI for MRgART should be able to focus not only on reliable segmentation but also on obtaining this segmentation in a few minutes to speed up the process of MRgART [16, 36].

Among the limitations of this study, the unbalanced percentage between males and females; some researchers investigated the influence of the relative position of the rectum and bladder on the mesorectum finding that due to anatomical differences, there are differences between males and females; on average half of the bladder of the female is located more cranially than the mesorectum for male patients. Therefore, a larger influence of bladder volume differences on the mesorectum can be expected for female patients compared to male patients [37].

Catheter-related bladder discomfort remains another limitation of the procedure; symptoms vary among patients from burning sensation and pain in the suprapubic and penile areas to urinary urgency, therefore some patients may refuse its use; some researchers have shown that bladder irritation due to muscarinic receptor-mediated involuntary contractions of bladder smooth muscle can also be caused by the mechanical stimulus of the urethral catheter [38].

The relatively small number of patients in this retrospective study and the lack of a multicenter study validation do not allow to generalise the results obtained, which need to be further investigated.

The use of an MR-linac offers the possibility of performing treatments of the highest quality, but the path is lined with obstacles that must be evaluated and managed in the best possible way [39]. These include recalculation using the sCT [40], the time factor that is a potential cause of the discrepancy in the final dosimetric result, but also the complexity of carrying out a quality treatment plan [41]. Artificial Intelligence (AI) is expected to rapidly contribute to MRgRT, primarily by safely and efficiently automating the various manual operations characterizing online adaptive treatments [36]. The use of a procedure within the clinical workflow to limit bladder changes during intra-fraction of MRgART, may be framed as an effective procedure that can contribute to excellent treatment.

## Conclusion

The use of a procedure to limit bladder changes during the online MRgART at MR-Linac is feasible and may contribute to ensure the accuracy of the treatment delivered.

## Data Availability

The data are fully available and will be included within the article.

## Abbreviations

CRT: Chemo-radiotherapy
pCR: Pathological complete response
LARC: Locally advanced rectal cancer
PTV: Planning target volume
CTV: Clinical target volume
GTV: Gross target volume
OAR: Organs at risk
MR-Linac: Magnetic resonance linear accelerators
MRI: Magnetic resonance imaging
MRgART: Magnetic resonance-guided adaptive radiotherapy
ED: Electronic density
CT: Computed tomography
sCT: Synthetic computed tomography
TPS: Treatment planning system
ATS: Adapt to shape
BfC: Bladder filling control
TPS: Treatment planning system
SCRT: Short course radiotherapy
DSC: Dice similarity coefficient
HD: Hausdorff distance
DVH: Dose volume histogram
